# Bacterial Cellulose as a Potential Bio-Scaffold for Effective Re-Epithelialization Therapy

**DOI:** 10.3390/pharmaceutics13101592

**Published:** 2021-09-30

**Authors:** Juin-Hong Cherng, Sheng-Chieh Chou, Chin-Li Chen, Yi-Wen Wang, Shu-Jen Chang, Gang-Yi Fan, Fang-Shiuan Leung, En Meng

**Affiliations:** 1 National Defense Medical Center, Graduate Institute of Life Sciences, Taipei 114, Taiwan; i72bb@mail.ndmctsgh.edu.tw; 2 Department and Graduate Institute of Biology and Anatomy, National Defense Medical Center, Taipei 114, Taiwan; christmas1035@ndmctsgh.edu.tw; 3 Department of Surgery, Division of Urology, Taoyuan Armed Forces General Hospital, Taoyuan 325, Taiwan; jay58042000@aftygh.gov.tw; 4 Department of Surgery, Division of Urology, Tri-Service General Hospital, National Defense Medical Center, Taipei 114, Taiwan; iloveyou@mail.ndmctsgh.edu.tw; 5 Laboratory of Adult Stem Cell and Tissue Regeneration, National Defense Medical Center, Taipei 114, Taiwan; belle661011@gmail.com (S.-J.C.); loyc@mail.ndmctsgh.edu.tw (G.-Y.F.); 6 College of Biological Science, University of California, Davis, CA 95616, USA; fleung@ucdavis.edu; 7 Department and Graduate Institute of Biochemistry, National Defense Medical Center, Taipei 114, Taiwan

**Keywords:** bacterial cellulose, scaffold, wound healing, epithelialization, tissue regeneration

## Abstract

Currently, there are several therapeutic approaches available for wound injury management. However, a better understanding of the underlying mechanisms of how biomaterials affect cell behavior is needed to develop potential repair strategies. Bacterial cellulose (BC) is a bacteria-produced biopolymer with several advantageous qualities for skin tissue engineering. The aim here was to investigate BC-based scaffold on epithelial regeneration and wound healing by examining its effects on the expression of scavenger receptor-A (SR-A) and underlying macrophage behavior. Full-thickness skin wounds were generated on Sprague-Dawley rats and the healing of these wounds, with and without BC scaffolds, was examined over 14 days using Masson’s trichome staining. BC scaffolds displayed excellent in vitro biocompatibility, maintained the stemness function of cells and promoted keratinocyte differentiation of cells, which are vital in maintaining and restoring the injured epidermis. BC scaffolds also exhibited positive in vivo effects on the wound microenvironment, including improved skin extracellular matrix deposition and controlled excessive inflammation by reduction of SR-A expression. Furthermore, BC scaffold significantly enhanced epithelialization by stimulating the balance of M1/M2 macrophage re-programming for beneficial tissue repair relative to that of collagen material. These findings suggest that BC-based materials are promising products for skin injury repair.

## 1. Introduction

The skin is the largest organ of the human body and provides a protective barrier against harmful external agents. Recently, biomaterials and tissue engineering have received a lot of attention with the focus on developing appropriate treatments for destructive skin injuries [1,2]. These strategies have been demonstrated to improve skin wound repair by reducing dehydration and infection, supporting vascularization and attracting matrix components, as well as sending cues to cells present in the local wound site [3,4,5,6]. In addition, biomaterials exhibit high biocompatibility, excellent permeability and non-toxicity, as well as tunable physical, morphological and mechanical properties. With these useful properties, functionalized biomaterials can promote wound healing by modulating local inflammation, ensuring selective cell infiltration and regulating the replication of various beneficial growth factors, cytokines and/or enzymes [7,8]. Although many studies have been conducted on the construction of properly engineered biomaterials, there are several limitations that prevent significant progress, including a poor understanding of cell–biomaterial interactions and host immune rejection. Thus, more comprehensive information is needed to understand the underlying mechanisms of how biomaterials affect cell behavior.

Choosing a suitable biomaterial is essential for the development of functionally engineered tissues. Recently, there has been increasing interest in using natural polymers such as collagen, cellulose, gelatin, plasma-based fibrin, keratin, silk and chitosan—separately or in combination—because they comprise protein motifs and/or bioactive molecules that naturally mimic the skin extracellular matrix (ECM) [5,9,10]. Constructing scaffold-like ECM is desirable as the ECM facilitates support to cell structure and functions, accelerating tissue formation and remodeling [11,12].

Cellulose is well-known as the most abundant natural polymer available and has been utilized for a long time to produce diverse products, ranging from industrial to medical applications, such as fibers for textiles, food ingredients and packaging, and hemodialysis and blotting membranes [13,14]. Cellulose occurs naturally in plants, consisting of β-1,4-glycosidic bonds with high crystallinity, indicating its resistance to depolymerization [15]. Bacterial cellulose (BC) is a biocompatible extracellular polysaccharide produced and secreted by some bacteria, such as the aerobic Gram-negative bacterium *Acetobacter xylinum* [16]. As BC is free of lignin and hemicellulose, it has unique properties, including chemical purity and a low energy synthesis process, although it shares the same molecular formula as plant celluloses. In addition, BC has excellent biodegradability and biocompatibility [17]. BC is also a remarkable biomaterial with tailor-made properties for various applications owing to its abundant reactive group structure that provides many possible modifications [18]. BC has been mainly applied as a scaffold for biomedical applications, including wound dressing [19], nerve regeneration [20], dental implants [21], cartilage growth [22] and vascular grafts [23]. In skin repair, BC shows good cytocompatibility, maintains a constantly moist environment, enhances exudate absorption, provides a highly porous, biocompatible and biodegradable architecture that mimics the ECM of skin and promotes tissue regeneration [24,25].

In wound repair, epithelialization is an essential parameter of the healing process, ensuring successful wound closure. Macrophages, key regulators of wound healing, play vital roles in all phases of repair by secreting various cytokines and chemokines associated with the production of pro-inflammatory mediators, angiogenesis and epithelialization [26]. Therefore, in this study, we aimed to investigate BC-based scaffold on epithelial regeneration and wound healing by examining its effects on the expression of scavenger receptor-A (SR-A) and underlying macrophage behavior. A better understanding of the epithelialization mechanism may lead to the development of significant therapeutic approaches with excellent healing potential.

## 2. Materials and Methods

### 2.1. Fabrication of Bacterial Cellulose (BC) Scaffold

BC (BF10005; The Far Eastern Group, Taipei, Taiwan (R.O.C.)) was produced by growing *Acetobacter xylinum* in a medium composed of buffered Hestrin–Schramm broth. After 2 days of incubation, the BC produced was soaked in coconut water (pH 4.0–4.4) at 30 °C for an additional 2 days. To remove the bacteria, BC fibers were washed with a 0.1 M NaOH solution at 90–95 °C followed by 0.25% H_2_O_2_ bleaching at 45 °C for 30 min. Finally, the BC fibers were washed several times in water until a neutral pH was reached. Subsequently, the BC fibers were compressed into a sheet and dehydrated with acetone (water content < 15%) with a thickness of 2 mm.

### 2.2. Characterization of Bacterial Cellulose (BC) Scaffold

#### 2.2.1. Scanning Electron Microscopy (SEM)

Briefly, sections of BC scaffold were attached to carbon stubs followed by gold coating using a sputter coating machine. A scanning electron microscope (HITACHI S-3000N, Hitachi High Technologies, Krefeld, Germany) at 1.5 kV accelerating voltage was used to observe the surface morphology of the scaffold.

#### 2.2.2. Fourier-Transform Infrared (FTIR) Spectroscopy

BC scaffold spectra were examined using FTIR spectroscopy (Nicolet 8700 spectrometer; Thermo Fisher Scientific, Waltham, MA, USA) equipped with the attenuated total reflectance (ATR) accessory and mercury–cadmium–telluride for infrared detection. Spectra were collected over a wavenumber range of 4000–500 cm^−1^ with a resolution of 1 cm^−1^. A total of 16 scans was produced.

### 2.3. Cell Culture

Isolation of human adipose stem cells (hASCs) was performed as previously described [27]. The tissue sample collection method was certified by the Institutional Review Board of Tri-Service General Hospital, Taipei, Taiwan (R.O.C.) (IRB 2-105-05-150). The primary culture of hASCs (provided by Dr. Cherng) was cultured in keratinocyte serum-free medium (KSFM, Life Technologies Ltd., Paisley, Scotland, UK) containing 10% fetal bovine serum (FBS; Hyclone, Logan, UT, USA) in a humidified 5% CO_2_ atmosphere at 37 °C.

RAW 264.7 cells (ATCC, Rockville, MD, USA) were maintained in Dulbecco’s modified Eagle medium (DMEM; Life Technologies, Carlsbad, CA, USA) containing 10% FBS (Life Technologies, Carlsbad, CA, USA) and 1% antibiotics (penicillin/streptomycin; Thermo Fisher Scientific, Waltham, MA, USA) at 37 °C under 5% CO_2_. The medium was renewed twice per week.

### 2.4. In Vitro Biocompatibility

The BC scaffold was sterilized by ultraviolet irradiation for 18 h, then seeded with hASCs at a density of 10^6^ cells/cm^2^ and incubated at 37 °C in a humidified 5% CO_2_ atmosphere. The culture medium was replaced every 2 days. The biocompatibility of the BC scaffold was investigated by trypan blue (Sigma-Aldrich; Merck, Darmstadt, Germany) staining. Briefly, the BC-hASC scaffolds were soaked in a 0.1% acetic acid solution for 30 min, followed by two phosphate buffered saline (PBS) washes and overnight refrigeration at 4 °C. The scaffolds were then post-fixed for 3 days in a 4% paraformaldehyde-sucrose solution, followed by rinsing with distilled water before staining with trypan blue solution for 10 min at 27 °C. The samples were washed twice with PBS to completely remove any remaining trypan blue residue before examination under a light microscope. Images were captured using an SPOT-RT digital camera (Diagnostic Instruments, Detroit, MI, USA).

### 2.5. Immunofluorescence Staining

After fixation, the samples were treated with 0.2% Triton X-100 for 30 min, followed by three 5 min washes with PBS. A 10% normal goat serum (Vector Laboratories Ltd., Burlingame, CA, USA) was used to block the samples, followed by incubation with primary antibodies (1:500 dilution), including rabbit polyclonal anti-octamer-binding protein 4 (anti-OCT-4), mouse monoclonal anti-nestin, mouse monoclonal anti-sex determining region Y-box 9 (anti-SOX-9) and mouse monoclonal anti SR-A (all from Santa Cruz Laboratories, Dallas, TX, USA), for 2 h at 27 °C. After three 5 min washes with PBS, secondary antibodies, including rabbit monoclonal anti-fluorescein isothiocyanate (1:1000 dilution; Jackson ImmuoResearch, West Grove, PA, USA) and mouse monoclonal anti-rhodamine (1:1000; AnaSpec, Fremont, CA, USA), were incubated with the samples for 1 h at 27 °C. Hoechst 33,342 (1:5000; AnaSpec, Fremont, CA, USA) was then added to the samples for 15 min for nuclei visualization. Finally, fluorescent images were observed using an inverted fluorescent microscope (Axio Lab.A1; Carl Zeiss AG, Oberkochen, Germany) equipped with a camera (Zeiss AxioCam ICm1; Carl Zeiss AG, Oberkochen, Germany). Semi-quantitative measurements of positive SR-A stained cells were conducted using the ImageJ software (National Institutes of Health, Bethesda, MD, USA).

### 2.6. Raw 264.7 Cells Cultured on BC and Collagen Scaffolds

A collagen scaffold was prepared as previously described [28]. Pig collagen (FlexiCol^®^ Sigma-Aldrich, Merck, Darmstadt, Germany) was dissolved in 1% (*v*/*v*) acetic acid and 0.275 mL of this solution was then loaded into glass vials. The solution was cooled to −20 °C and stored overnight before being transferred into a freeze-drying chamber (FD12-2S; Kingming, Taipei, Taiwan) and dehydrated under a 300 mbar vacuum at −45 °C for 24 h. This process was evenly ramped to 25 °C, followed by incubation in a 2.5% (*w*/*v*) polycaprolactone/dichloromethane solution for 30 min in a closed vial. The solvent was allowed to evaporate in air before adding RAW 264.7 cells (10^5^ cells/mL) onto the BC and collagen scaffolds. The scaffolds were then incubated in culture medium for 24 h in a humidified 5% CO_2_ atmosphere at 37 °C.

### 2.7. Rat Model of a Surgical Epidermal Defect

A total of 30 male Sprague-Dawley rats (250–300 g; Bio-LASCO Co. Ltd., Taipei, Taiwan (R.O.C.)) were used to develop the skin epidermal defect model. All animal protocols were approved by the Institutional Animal Care and Use Committee (IACUC-17-068) at the Animal Center of the National Defense Medical Center, Taipei, Taiwan, R.O.C. The rats were randomly divided into a BC scaffold treatment group and a control lesion group. Briefly, the rats were anesthetized with an intraperitoneal injection of xylazine (8 mg/kg) and ketamine (100 mg/kg). Two full-thickness skin wounds, 1 cm in diameter, were generated using a surgical blade on the dorsum of each rat. The wounds were then covered with BC scaffold for the experimental group and left untreated and covered with plain medical gauze for the control lesion group. The rats were kept in individual cages and euthanized on day 7 or day 14 after wound generation by intraperitoneal administration of an overdose of sodium pentobarbital (≥100 mg/kg). The wound tissues were harvested and immediately stored in 10% formalin with sucrose for further analysis.

### 2.8. Masson’s Trichrome Staining

Briefly, 30 μm cryo-sectioned tissue samples were fixed with 4% paraformaldehyde. The samples were then stained using Masson’s trichrome solution (Sigma-Aldrich, Laborchemikalien GmbH, Hanover, Germany) according to the manufacturer’s instructions. An inverted microscope (BX53; Olympus, Tokyo, Japan) was used to observe the samples.

### 2.9. Multiplex Protein Biomarker Immunoassays

Cytokine and chemokine production in the supernatant of cultured RAW 264.7 cells were quantified using the Milliplex MAP Mouse Cytokine/Chemokine Panel (Merck Millipore, Darmstadt, Germany) following the manufacturer’s instruction. Medium from scaffold-treated and control cells was used and granulocyte-macrophage colony-stimulating factor (GM-CSF), macrophage colony-stimulating factor (MCSF), interferon-gamma (IFN-γ), interleukin-1 alpha (IL-1α), interleukin-1 beta (IL-1β), interleukin-2 (IL-2), interleukin-4 (IL-4), interleukin-10 (IL-10), interleukin-12(p70) (IL-12(p70)), interleukin-17 (IL-17), chemokine (C-X-C motif) ligand 1 (CXCL-1), macrophage inflammatory protein-1 alpha (MIP-1a), macrophage inflammatory protein-1 beta (MIP-1b), regulated on activation, normal t expressed and secreted (RANTES), tumor necrosis factor-alpha (TNF-α), nuclear factor kappa B (NF-κB) and protein kinase B (Akt) were analyzed. For the Milliplex assay, beads and the appropriate detection antibodies were added to the control or treated cell lysates, which were then incubated with antibody-conjugated magnetic beads overnight at 4 °C. The concentration of recovered bead complexes was read on a Magpix Multiplex Platform (Luminex Corporation, Austin, TX, USA). Median fluorescent values were recorded from a minimum of 80 beads that were used for data analysis. Standard curves and data were analyzed using the Milliplex Belysa™Immunoassay Curve Fitting Software (Version 1.1, Merck KGaA, Darmstadt, Germany).

### 2.10. Quantitative Real Time Polymerase Chain Reaction (qRT-PCR)

Briefly, RNA was isolated from RAW 264.7 cells cultured on BC and collagen scaffolds. The quantity and purity of isolated RNA were assessed using a NanoDrop™ 2000 Spectrophotometer (Thermo Fisher Scientific, Waltham, MA, USA). cDNA was generated using the High-Capacity cDNA Reverse Transcription Kit (Applied Biosystems, Waltham, MA, USA). qRT-PCR was carried out using the LightCycler^®^ 480 SYBR Green I Master (Roche Life Science, Penzberg, Germany). Briefly, the reaction included an initial activation step at 95 °C for 3 min, 40 cycles of denaturation at 95 °C for 5 s, annealing/extension at 60 °C for 20 s and, lastly, a high-resolution melting curve analysis from 65–95 °C with 0.5 °C increments, 5 s per step. Equal amounts of cDNA were added for all samples, which were run on a LightCycler ^®®^ 480 System (Roche Life Science, Penzberg, Germany). All measurements were based on quadruplicate measurements of each cell culture condition and normalized to the internal reference gene, glyceraldehyde 3-phosphate dehydrogenase (GAPDH). The comparative cycle threshold (DDCT) method was used to calculate the relative fold-changes. Primers were designed using the Primer-BLAST tool (National Center for Biotechnology Information, National Institutes of Health, Bethesda, MD, USA) as follows: mouse iNOS-Forward, CCA AGC CCT CAC CTA CTT CC and iNOS-Reverse, CTC TGA GGG CTG ACA CAA GG; mouse Arg-1-Forward, CAT GGG CAA CCT GTG TCC TT and Arg-1-Reverse, TCC TGG TAC ATC TGG GAA CTT TC.

### 2.11. Statistical Analysis

The statistical analysis was performed using the Statistical Package for Social Sciences version 18 (SPSS, Chicago, IL, USA). The data are presented as mean ± standard error of the mean and data means were compared using a one-way ANOVA. Data were considered statistically significant at *p* < 0.05.

## 3. Results and Discussion

### 3.1. Characteristics of Bacterial Cellulose Scaffold

**Figure 1:**
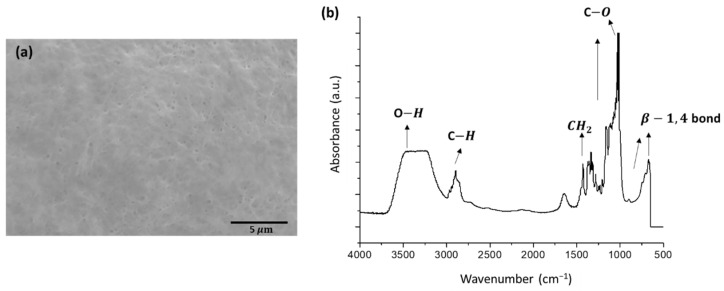
Characteristics of bacterial cellulose scaffold. (**a**) Scanning electron microscopy. (**b**) Fourier-transform infrared spectroscopic analysis.

In skin tissue engineering, the functionality of tissue repair depends on the microstructure of the applied scaffold. Appropriate scaffolds play key roles in the regeneration of skin tissues by providing a suitable platform and supplying various factors related to the proliferation, differentiation and survival of cells [29]. SEM analysis demonstrated that the surface of the BC scaffold was smooth, comprised of ultrafine BC fibrils with a pore diameter of ~0.2 μm, supporting substance interchange and cell adhesion (Figure 1a). The functional groups of the BC scaffold were further examined by FTIR spectroscopy (Figure 1b). The FTIR spectrum displayed strong peaks for structures, including β-1,4 bond vibrations (675–890 cm^−1^), stretching vibrations of the antisymmetric C–O–C bridge and symmetric C–O groups (1000–1300 cm^−1^), CH_2_ bending relating to crystalline/amorphous proportions in cellulosic molecules (1432 cm^−1^), C–H groups (2892 cm^−1^) and stretching vibrations of the OH groups (3200–3500 cm^−1^), which implies a relative abundance of cellulose Iα [30,31]. Similar results have also been reported in previous studies, indicating that A. xylinum can produce typical cellulose [32,33].

### 3.2. In Vitro Biocompatibility of Bacterial Cellulose Scaffold

**Figure 2:**
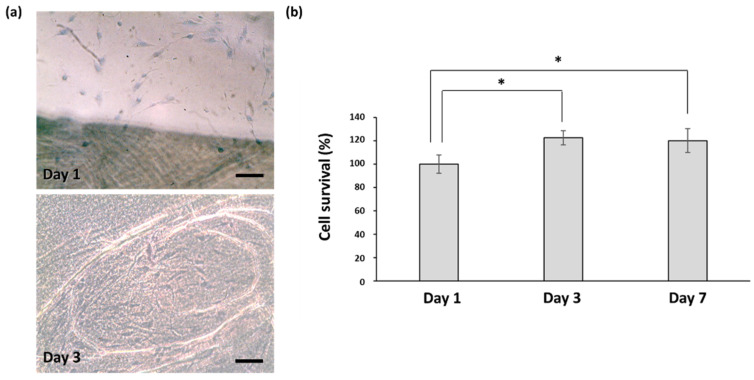
Study of in vitro biocompatibility of bacterial cellulose scaffold incubated with human adipose stem cells (hASCs) using trypan blue staining. (**a**) Growth of hASCs with time. (**b**) Quantification of cell survival (scale bar = 200 µm; * *p* < 0.05).

**Figure 3:**
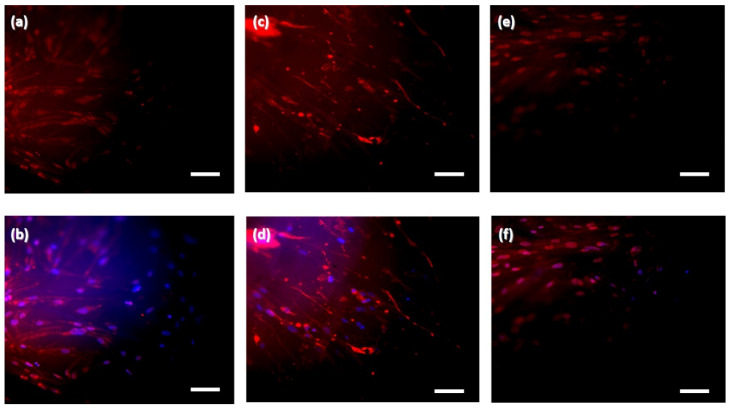
Immunofluorescence staining of human adipose stem cells cultured on bacterial cellulose scaffold for 14 days. (**a**,**b**) Octamer-binding protein 4 (OCT-4) and OCT-4/Hoechst overlay expression, respectively. (**c**,**d**) Nestin and Nestin/Hoechst overlay expression, respectively. (**e**,**f**) Sex determining region Y-box 9 (SOX-9) and SOX-9/Hoechst overlay expression, respectively (scale bar = 200 µm).

Biocompatibility is an important feature of scaffolds that provides conducive temporary platforms for successful cell–biomaterial interactions and tissue formation. To evaluate the effects of the BC scaffold on the cellular microenvironment, we incubated the scaffold with hASCs and used trypan blue staining to observe their proliferation. As shown in Figure 2a, the BC scaffold promoted cell growth remarkably after 3 days and significant cell survival after 7 days of incubation (Figure 2b), proving its excellent biocompatibility. It is suggested that BC scaffolds enhance cell adhesion owing to their crystalline fibrillar structure, which creates a large surface area. Proper cell adhesion that initiates cytoskeletal protein mediation is favorable for the completion of cell mitosis [34]. We further evaluated the differentiation potential of hASCs cultured on BC scaffolds using several markers, including OCT-4, nestin (stemness differentiation marker) and SOX-9 (a keratinocyte differentiation marker) [35,36]. After 14 days incubation, there was a positive expression of OCT-4 (Figure 3a,b) and nestin (Figure 3c,d) by hASCs on the BC scaffold, indicating that BC can maintain the stemness function of cells. This mechanism is critical for physiological tissue renewal and regeneration after injury [37]. In addition, the positive expression of SOX-9 (Figure 3e,f) in hASCs on BC scaffolds showed that BC has the potential to promote keratinocyte differentiation of hASCs. Many studies have demonstrated that mesenchymal stem cells can participate in wound re-epithelialization by differentiating into keratinocytes, thus regenerating the skin epidermis [36,38,39,40]. Keratinocytes are vital in maintaining the epidermis and restoring it after injury [41].

### 3.3. In Vivo Assessment Using a Rat Skin Defect Model

**Figure 4:**
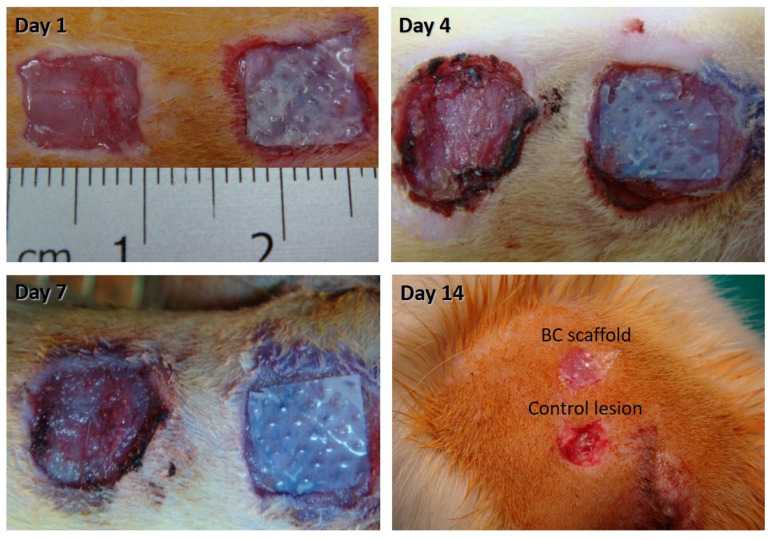
Rat model of skin defects healing over time without (**left side**) or with bacterial cellulose scaffold covering (**right side**).

To evaluate the effects of the BC scaffold on epithelial regeneration in vivo, we developed a rat skin defect model, which was either treated with BC scaffold or remained empty as a control lesion for up to 14 days. Representative images of wound healing over time are shown in Figure 4. Compared to the control lesion, the wound site with BC scaffold treatment was smaller and surrounded by smooth tissue without redness. However, both skin defects demonstrated ongoing wound closure.

**Figure 5:**
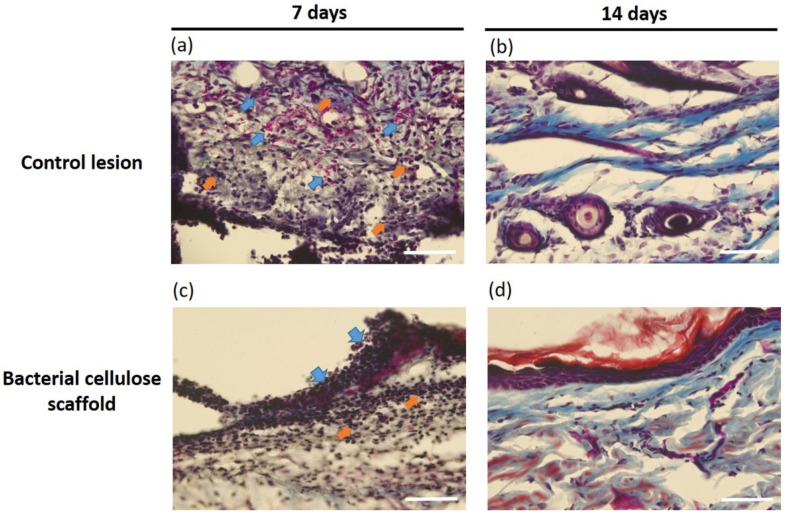
Histological analysis of rat skin defects using Masson’s trichrome staining. (**a**,**b**) Control lesion group after 7 and 14 days of treatment, respectively. (**c**,**d**) Bacterial cellulose scaffold group after 7 and 14 days of treatment, respectively (blue arrow = immune cell infiltration; orange arrow = granulation tissue; scale bar = 50 µm).

A qualitative assessment is necessary to determine whether there are observable tissue, cellular, or molecular differences caused by the treatment during the wound healing process. Dermal wound repair involves dynamic activities, including interaction between epidermal and dermal cells and angiogenesis, as well as ECM and plasma-derived proteins for successful epithelialization [42]. In the absence of re-epithelialization, failed wound repair occurs. To evaluate the quality of epithelial regeneration in the wound tissue, a histological analysis using Masson’s trichrome staining was performed (Figure 5). The results revealed that re-epithelialization of the wound was occurring with BC scaffold treatment. Compared to the untreated control lesion that showed a less clear structure, more empty spaces, hair follicle damage and high numbers of neutrophilic inflammatory cells (Figure 5a), wounds treated with BC scaffolds showed increased healing effects, as indicated by the presence of keratinocytes and fewer granular cells or inflammatory cells after 7 days (Figure 5c). During re-epithelialization, fibroblasts are recruited to rebuild the dermal layer along with the migration of keratinocytes from the edges of the wound to re-epithelialize the surrounding matrix [43]. After 14 days of wound healing, in this study, an improved arrangement of collagen fibers, stratified squamous epithelium and dense newborn subcutaneous tissue, which integrated with the granular tissue in the epidermis, was displayed in wound tissue with BC scaffold treatment (Figure 5d), when compared with the untreated lesion control (Figure 5b). Previous studies reported that BC applied as wound dressing demonstrates good cytocompatibility and histocompatibility with no fibrotic tissues around the implants and showed better tissue regeneration and faster healing in the in vivo studies performed on a large area of skin [44,45,46]. Overall, the results reflect the positive role of BC in ECM deposition and re-epithelialization of wounds.

**Figure 6:**
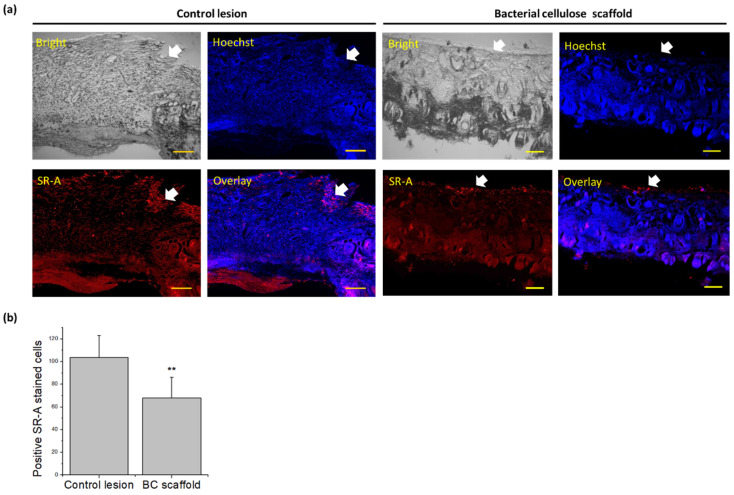
Immunostaining of scavenger receptor-A (SR-A) expression in rat skin defects after 7 days of treatment. (**a**) Control lesion compared with bacterial cellulose (BC) scaffold. (**b**) Semi-quantitative analysis of SR-A positively-stained cells using ImageJ (scale bar = 200 µm; ** *p* < 0.01).

The beneficial re-epithelialization shown by the BC scaffold could be due to the reduction in the excessive immune response of macrophages, which would avoid persistent inflammation and tissue damage. In this study, we evaluated the expression of SR-A at the wound sites after 7 days of treatment (Figure 6). SR-A is a membrane-bound receptor that is relatively abundant under oxidative stress conditions in macrophages, vascular smooth muscle and endothelial tissues [47]. In macrophages, SR-A participates in the identification and removal of pathogens. However, SR-A1-null macrophages exhibit elevated pro-inflammatory responses, such as increased p42/44 MAPK phosphorylation, NF-κB nuclear translocation and increased secretion of TNFα, IL-6 and IFNβ [48]. As shown in Figure 6a, the relative expression of the SR-A at the wound site was weaker in the BC scaffold treatment than in the control lesion, indicating that the activity of macrophages was downregulated along with the formation of integrated keratinocytes in the superior epithelial tissue. Semi-quantitative analysis revealed a significantly lower number of SR-A positively stained cells in the BC scaffold group than in the control lesion group (*p* < 0.01; Figure 6b). Preclinical and clinical studies have shown that a reduced inflammatory response and improved epithelialization during treatment are associated with better wound closure and scar histology [49,50]. Based on this result, we further evaluated the underlying mechanism of BC scaffold treatment on macrophage behavior.

### 3.4. Immunoregulation of Bacterial Cellulose Scaffold on Macrophage Behavior

**Figure 7:**
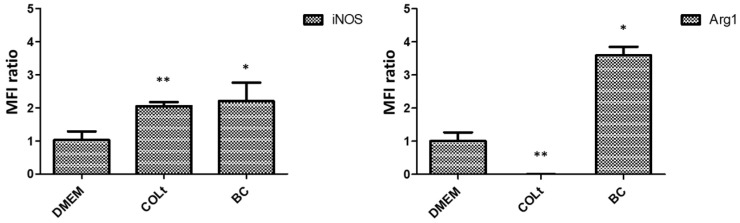
Expression of inducible nitric oxide synthase (iNOS) and arginase-1 (Arg1) secreted by macrophages cultured on bacterial cellulose (BC) scaffold, collagen total type material (COLt), or in DMEM for 24 h (* *p* < 0.05; ** *p* < 0.01).

As the expression of SR-A was up-regulated in the epidermis of rats with skin defects, we investigated the immunoregulation of BC on macrophages to define their M1/M2 behavior. We hypothesized that BC could stimulate the balance of M1/M2 macrophage reprogramming, which is essential for promoting cell proliferation and tissue repair. In this study, we compared the effects of BC scaffold and collagen total type material (COLt) on M1/M2 macrophage phenotypes by analyzing the expression of iNOS and Arg-1, as well as M1/M2 macrophage-related cytokines. Collagen, one of the primary components of ECM, is commonly used as a dressing-based material in wound healing because it can promote angiogenesis and re-epithelialization of wound tissue [51]. In the subsequent experiment, we compared BC scaffold effectiveness to that of collagen-based material, which served as a control dressing, to explore any differences in the regulation of the skin wound inflammation response. The results showed that BC scaffold significantly enhanced the expression of iNOS and Arg-1, compared to that of COLt (*p* < 0.05; Figure 7). iNOS is induced by M1 macrophages, which have a pro-inflammatory phenotype with pathogen-killing properties, while Arg-1 is induced by M2 macrophages, which support cell proliferation and tissue repair [52,53]. In relation to SR-A expression, the expression of Arg-1-induced M2 was higher than that of iNOS-induced M1 in the BC culture. The role of SR-A in triggering pro-inflammatory reactions is associated with phagocytosis and uptake of waste products [54]. One study revealed that SR-A1 antibody pre-treatment increased IL-10 expression in RAW264.7 cells. This indicates that SR-A1 facilitates the inflammatory response not only by inducing the secretion of pro-inflammatory cytokines, but also by suppressing the expression of anti-inflammatory cytokines, which was accompanied by a decrease in M1 macrophages and an increase in M2 macrophages [55]. Our results suggest that BC is not only able to control the inflammation response, but it also functions in M1/M2 polarization.

**Figure 8:**
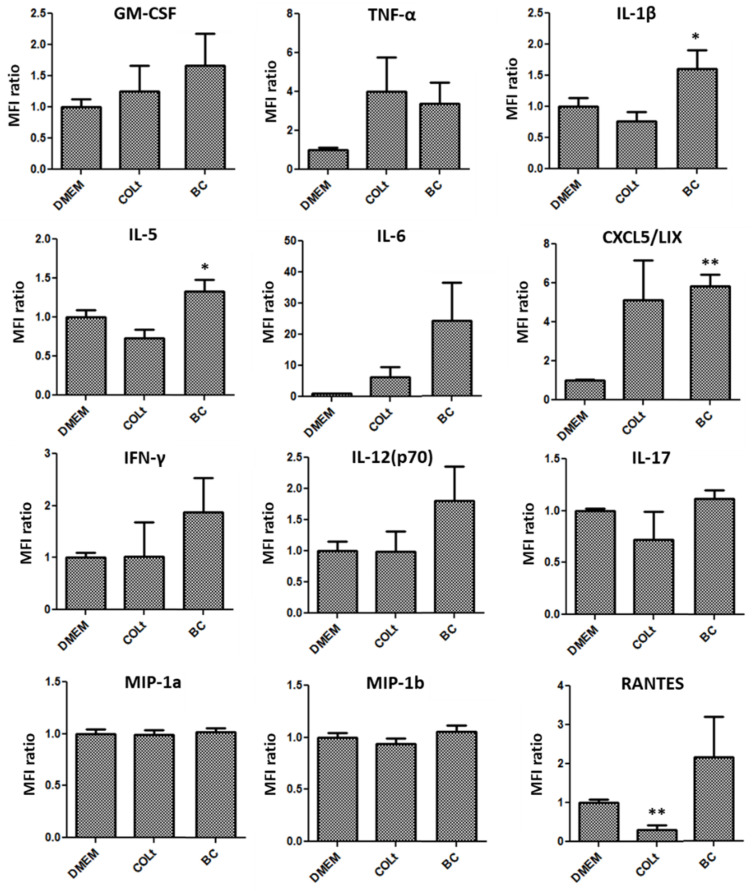
Expression of M1 macrophage-related cytokines secreted by macrophages cultured on bacterial cellulose (BC) scaffold, collagen total type material (COLt), or in DMEM for 24 h (GM-CSF = granulocyte-macrophage colony-stimulating factor; TNF-α = tumor necrosis factor-alpha; IL-1β = interleukin-1 beta; IL-5 = interleukin-5; IL-6 = interleukin-6; CXCL5/LIX = chemokine (C-X-C motif) ligand 5/lipopolysaccharide-induced chemokine; IFN-γ = interferon-gamma; IL-12(p70) = interleukin-12(p70); IL-17 = interleukin-17; MIP1a = macrophage inflammatory protein-1 alpha; MIP1b = macrophage inflammatory protein-1 beta; RANTES = regulated on activation, normal t expressed and secreted; * *p* < 0.05; ** *p* < 0.01).

**Figure 9:**
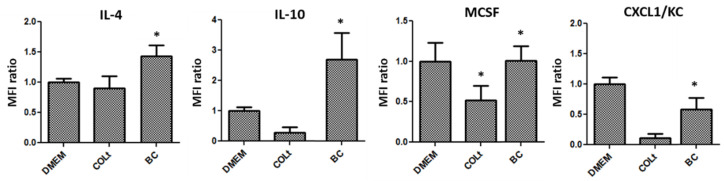
Expression of M2 macrophage-related cytokines secreted by macrophages cultured on bacterial cellulose (BC) scaffold, collagen total type material (COLt), or in DMEM for 24 h (IL-4 = interleukin-4; IL-10 = interleukin-10; MCSF = macrophage colony-stimulating factor; CXCL1/KC = chemokine (C-X-C motif) ligand 1/keratinocyte-derived chemokine; * *p* < 0.05).

Cytokine assay results demonstrated that culturing macrophages with BC elevated the levels of M1 macrophage-related cytokines, including GM-CSF, TNF-α, IL-1β, IL-5, IL-6, CXCL5/LIX, IFN-γ, IL-12(p70), IL-17, MIP1a, MIP1b and RANTES, compared with that of COLt (Figure 8). The expression of M1 macrophage-related cytokines in COLt was not significantly different from that of the control DMEM group, indicating collagen may not activate macrophage polarization. In addition, BC scaffold had the highest proportion of M2 macrophage-related cytokines, such as IL-4, IL-10, MCSF and CXCL1/KC (Figure 9).

Macrophages play vital roles in the phases of wound healing by circulating into target tissues and differentiating into polarized M1 and M2 macrophages, as influenced by microenvironment signals [56]. Understanding macrophage activity is important for tissue repair and homeostasis maintenance. M1 macrophages are stimulated by Th1 cytokine IFN-γ and bacterial components (e.g., lipopolysaccharide and peptidoglycan), while M2 macrophages are stimulated by different stimuli, including IL-4, IL-10 and IL-13 [57,58]. During the wound healing process, M1 macrophages are active in pro-inflammatory and antimicrobial responses by producing various cytokines and chemokines, including TNF-α, IL-1, IL-6, IL-12, IFN, CXCL1–3, CXCL5 and CXCL8–10. In contrast, M2 macrophages occur in response to IL-4, IL-10, IL-13, IL-33 and TGF-β signals [59,60].

**Figure 10:**
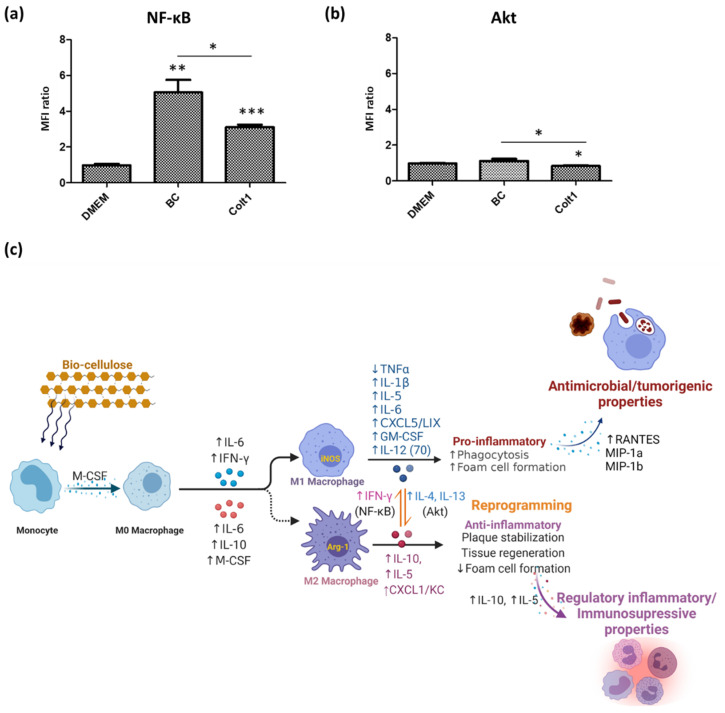
Signaling pathways alteration in macrophages affected by bacterial cellulose (BC) scaffold after 24 h of treatment. (**a**) Nuclear factor kappa B (NF-κB). (**b**) Protein kinase B (Akt). (**c**) Summary of macrophage polarization states (this figure was created with BioRender.com and based on icons from biorender.com). (* *p* < 0.05; ** *p* < 0.01; *** *p* < 0.001).

Consequently, macrophages also play key roles in immunity. A balance between M1 and M2 macrophages can thus differentially regulate beneficial tissue repair in inflammatory diseases [61,62]. However, macrophage phenotypes are still alterable in response to microenvironment stimuli; proinflammatory stimuli can trigger a switch in phenotype towards the anti-inflammatory M2 phenotype. In turn, anti-inflammatory stimuli can trigger a switch in phenotype towards the proinflammatory M1 phenotype [63]. In this study, we investigated the shifting of macrophage phenotypes in response to scaffold treatment. Macrophage phenotype shifting affects synchronized changes in signaling pathway activities, such as JNK, PI3K/Akt, Notch, JAK/STAT, TGF-β and TLR/NF-κB. Furthermore, both phenotypes can tolerate reversible functional changes that lead to macrophage plasticity and the ability to be reprogrammed given the proper stimuli [64,65]. Figure 10a demonstrated that BC scaffold significantly enhanced the activation of the NF-κB pathway in IFN-γ-stimulated macrophages. The NF-κB pathway is primarily involved in immune and inflammatory regulation by modulating pro-inflammatory mediators and cytokines associated with inflammation, apoptosis and proliferation [66]. NF-κB pathway activation is also vital for M1 macrophage polarization and its pro-inflammatory effects [67]. Furthermore, the switching phenotype may be involved in the several isoforms of Akt, allowing macrophages to be reprogrammed to both M1 and M2 phenotypes in response to the same LPS ligand via the PI3K/Akt-signaling pathway [68]. As shown in Figure 10b, BC scaffold significantly elevated the activation of the Akt signaling pathway. A study reported that the inhibition of the PI3K/Akt pathway can suppress M2 macrophage polarization [69]. Our data suggest that Akt1 ablation results in an M1 phenotype, while ablation of Akt2 results in an M2 phenotype. Additionally, in the BC scaffold culture condition, we found that, although the cytokines were simultaneously secreted by M1/M2 macrophages, the expression of M1 shift to M2 and M2 shift to M1 cytokines were also elevated, demonstrating a constantly dynamic homeostasis (Figure 10c). Moreover, the M1/M2 switching phenotype induces the production of M1 mediators in response to IFN-γ and, in response to IL-4 and IL-13, their phenotype induces the production of M2 mediators [70]. This continuum performs an essential role during inflammation; hence, it implies the ability of BC in helping to maintain the balance of M1/M2 macrophage reprogramming for beneficial tissue repair.

## 4. Conclusions

In summary, our study demonstrates the potential of BC as an effective bio-scaffold for skin tissue repair. The results suggest that a BC scaffold plays a positive role in the wound microenvironment, including improving skin ECM deposition, controlling excessive inflammation responses by reducing SR-A expression and enhancing the epithelialization process by stimulating the balance of M1/M2 macrophage reprogramming, all of which are beneficial in tissue repair. Together, these findings support the use of BC-based materials, such as surgical dressings or carriers, in cell therapy treatment for skin injury management.

## Data Availability

The data are available upon request.
